# The Lecanemab Clarity AD Open‐Label Extension in Early Alzheimer’s Disease: Initial Findings From the 48‐Month Analysis

**DOI:** 10.1002/alz70861_108905

**Published:** 2025-12-23

**Authors:** Christopher H van Dyck, David Li, Michio Kanekiyo, Shobha Dhadda, Steven Hersch, Michelle Gee, Michael C. Irizarry, Lynn D Kramer

**Affiliations:** ^1^ Yale School of Medicine, New Haven, CT USA; ^2^ Alzheimer's Disease Research Unit, Yale School of Medicine, New Haven, CT USA; ^3^ Eisai Inc., Nutley, NJ USA; ^4^ Eisai Ltd., Hatfield UK

## Abstract

**Background:**

Lecanemab is a humanized IgG1 monoclonal antibody that binds with high affinity to amyloid‐beta (Aβ) protofibrils. In the 18‐month, phase 3 Clarity AD study, lecanemab demonstrated amyloid reduction and cognition and function decline slowing in participants with early symptomatic Alzheimer’s disease (AD). An ongoing open‐label extension (OLE) of Clarity AD is evaluating long‐term safety and efficacy of lecanemab. Herein, we report the initial findings up to 48 months from the ongoing Clarity AD OLE study.

**Method:**

Clarity AD is an 18‐month, randomized study (Core) followed by an OLE phase in individuals with early AD. Clinical (CDR‐SB, ADAS‐Cog14, and ADCS‐MCI‐ADL) outcomes and safety were evaluated from OLE data out to 48 months. Continued lecanemab treatment in the OLE beyond the 18 months from the Core study was compared to a matched control from Alzheimer's Disease Neuroimaging Initiative (ADNI) data. Subgroup analyses were conducted for participants with no/low baseline tau. Efficacy assessments were also summarized as the percentage of participants who had ‘no decline’ or had ‘improvement’ from Core baseline at each timepoint.

**Result:**

Overall, 1734 participants were treated with lecanemab across the Core and OLE. Across clinical endpoints, lecanemab‐treated participants continued to benefit through 48 months. In the OLE, lecanemab treatment continued to delay progression through 48 months compared to ADNI matched control, with differences in CDR‐SB increasing over 18 months (0.52), 36 months (1.01), and 48 months (1.75). Consistent rates of clinical stability or improvements were observed across assessments regardless of baseline tau levels, with the highest rates of improvements observed for the no/low tau group at 48 months (CDR‐SB: no decline:69%, improvement:56%; ADAS‐Cog14: no decline:51%, improvement:51%; ADCS MCI‐ADL: no decline:64%, improvement:58%). No new safety signals were observed. ARIA rates were low and similar to ARIA rates on placebo after 6 months.

**Conclusion:**

In Clarity AD, the observed increasing treatment difference with ongoing lecanemab treatment in participants treated through 48 months versus ADNI matched placebo data is consistent with a durable disease‐modifying effect. The long‐term safety profile of lecanemab was confirmed, with no new safety signals.

